# Sentence Comprehension and Its Association with Executive Functions in Patients with Parkinson's Disease

**DOI:** 10.4061/2011/213983

**Published:** 2011-10-24

**Authors:** Katrien S. F. Colman, Janneke Koerts, Laurie A. Stowe, Klaus L. Leenders, Roelien Bastiaanse

**Affiliations:** ^1^Department of Neurolinguistics, University of Groningen, P.O. Box 716, 9700 AS Groningen, The Netherlands; ^2^Department of Clinical and Developmental Neuropsychology, University of Groningen, Grote Kruisstraat 2/1, 9712 TS Groningen, The Netherlands; ^3^Department of Neurology, University Medical Center Groningen, Hanzeplein 1, P.O. Box 30.001, 9700 RB Groningen, The Netherlands

## Abstract

Coexistent impairments in executive functions and language comprehension in patients with Parkinson's disease (PD) have been repeatedly observed. In this study, the aim was to provide insights into the interaction between linguistic representation and processing and executive functioning. Therefore, sentence comprehension and executive functions were assessed in 28 Dutch-speaking PD patients and 28 healthy control subjects. Three aspects of the sentence materials were varied: (1) phrase structure complexity, (2) sentence length, and (3) picture congruence. PD patients with mild-to-moderate disease severity showed decreased sentence comprehension compared to healthy control subjects. The difficulties encountered by PD patients were not limited to one aspect of the sentence materials. The same pattern of results was present in healthy control subjects. Deficits in set-switching were specifically associated with the comprehension of passive sentences. Generally, our study confirms that there does not appear to be a language faculty encapsulated from the influence of executive functions.

## 1. Introduction

In Parkinson's disease (PD), the components of the cortico-striato-cortical circuits are not in optimal interaction. Motor symptoms of tremor, bradykinesia, and rigidity are the clinical hallmark of the disease [1]. However, nonmotor symptoms are often present [2]. In particular, cognitive impairments in the domain of executive functioning have frequently been observed even in very early stages of PD [3]. Additionally, several independent researchers have demonstrated a sentence comprehension deficit in PD patients suggesting the involvement of the cortico-striato-cortical circuits in language processing. Since the early 1990s, a number of studies have revealed that long sentences and complex syntactic structures (i.e., noncanonical structures) are vulnerable in individuals with PD (see [4, 5] for a review). There is, however, no consensus concerning the *functional* basis of the sentence comprehension impairment in PD. Some authors attribute the sentence comprehension deficit to an impairment of grammatical processing as such [6–9]. This viewpoint suggests that linguistic deficits in PD will affect specific aspects of language structure, as in aphasia. 

Other researchers, however, suggest that executive dysfunction(s) are the functional basis of the sentence comprehension difficulties in PD. This viewpoint suggests that language processing deficits in PD are always associated with executive function deficits. Under this latter view, the language faculty is not totally modular in nature but depends on other cognitive functions. For instance, comprehending a sentence demands that listeners flexibly guide their attention to relevant linguistic information, maintain information in working memory during the incremental development of the sentence interpretation, and inhibit prepotent or incorrect parsing. 

However, which aspect(s) of executive function are most important for language comprehension is under debate. The underlying cognitive impairment responsible for sentence comprehension deficits in PD has been claimed to be in (1) set switching (2) sequencing [10–16], (3) inhibition [17, 18], (4) information processing speed [19–21], or (5) verbal working memory capacity [22, 23]. In the current study, we will investigate the majority of these functions and their relationship to sentence comprehension in patients with PD. If grammatical processing and executive functions are not in fact encapsulated and separable mechanisms, then comprehension of syntactic structures is achieved by the influence of executive functions on language-specific knowledge. Under this view, executive dysfunction should correlate strongly with problems in sentence comprehension. 

The present study assessed sentence comprehension by manipulating phrase structure complexity of the sentences (active versus passive sentences) and sentence length (short versus long sentences). Additionally, trials differed with respect to whether or not a spoken sentence was congruent with a picture. Furthermore, executive functions hypothesized to be relevant to sentence comprehension including attention, set switching, inhibition, working memory, and abstract sequencing abilities were assessed. Based on earlier research, we expect to find a lower overall sentence comprehension score for the Dutch-speaking PD patients and strong correlations between sentence comprehension and one or more measures of executive function in patients with PD. The results of the present study will have implications for the modularity of these two aspects of cognition. 

PD patients have impairments in the comprehension of the syntactically complex passive sentences [24, 25] and longer sentences [21, 23]. Sentences are defined as syntactically complex when the thematic roles (such as “agent”—the one who is doing the action—and “theme”—the person or object that is undergoing the action) are not in their basic (or canonical) position and, therefore, require extra grammatical operations. For example, in a passive sentence like “*the cat is chased by the dog*”, the cat is the theme and the dog is the agent, whereas in English, the subject is usually the agent. In such a sentence, the thematic roles are in derived position (i.e., the theme is preceding the agent). 

Problems with passives in PD have been described when comprehension of the passive voice was complicated by additional factors such as length in combination with incongruence of the sentence with the picture [10], lack of cues from real-world knowledge to help in understanding the sentence [12], or when the passive sentences contained a center-embedded relative clause which is well known to increase processing demands [11, 12]. The impact of these additional factors suggests that the impairment is not necessarily to syntactic processing per se, as it is in aphasia. In addition to problems with passives, past research in PD has found deficits in comprehending sentences containing object-relative clauses (e.g., “*the girl that the boy kissed watched the movie*”), which are also noncanonical structures. These deficits correlated to impaired ability to shift between or inhibit competing cognitive sets, verbal working memory, and verbal fluency scores [18, 20]. 

Similarly, comprehension of passive sentences might rely on one or more executive function. First, impairments in *set shifting/set switching* are apparent in PD [3, 26]. During sentence comprehension, set-switching is necessary to disengage from the canonical (expected) thematic role assignment and increase activation of the noncanonical syntactic assignment. If this process relies on set shifting deficits in the comprehension of passive sentences should be associated with deficits in set shifting in PD. However, set-shifting strongly depends on the ability to inhibit the irrelevant set [12]. It is, therefore, possible that *inhibition* is correlated with the comprehension of passive sentences. Third, *working memory* might play a role in the comprehension of passive sentences since comprehension relies on the ability to keep words and phrases in memory and manipulate them to understand who is doing what to whom, particularly in noncanonical and multiple embedded sentences [27, 28]. Finally, *sequencing* abilities may be related to the comprehension of passive sentences, since sentence comprehension is a time- and order-related phenomenon [29, 30]. Since PD patients are known to have problems with sequencing of actions [31] complex sequencing abilities may well also be related to the comprehension of syntactically more complex passive sentences. 

As well as canonicity of thematic role assignment, length appears to be relevant to the relationship between sentence comprehension and executive functioning [21, 23]. Grossman et al. [21] addressed working memory during the comprehension of sentences by including three words (short) or seven words (long) at a particular position in the sentences. In their study the striatum, which is dysfunctional in patients with PD, appeared to be related to the comprehension of long sentences, relative to short sentences. Skeel et al. [23] contrasted semantically loaded sentences and syntactically loaded sentences of several levels of difficulty. The semantic difficulty was manipulated by increasing the number of semantic elements. This study demonstrated that PD patients showed deficits in the processing of both semantically and syntactically loaded sentences but showed no working memory impairments.

The length manipulation of the sentences in the present study is somewhat different from the one used in the Grossman et al. [21] and the Skeel et al. [23] study. The present length manipulation involved the inclusion of adjectives before the agent noun, the theme noun, and in a prepositional phrase (PP) (see sentence (2) in Section 2 for an example of the length manipulation). In other words, our length manipulation, involved an accumulation of information relative to the short, “bare” sentences which presumably puts an extra load on short-term memory [32, 33]. If this executive function is important for language processing, short-term memory deficits will be associated with difficulties in processing of long sentences, independent of the syntactic complexity of the sentence. 

Finally, sentence comprehension in the context of a task, regardless of various factors which add to complexity, needs to be considered. The ability to understand sentences to carry out a task depends on the level of sustained attention (visual or auditory). Sustained attention is attention which remains focused over a longer period of time; this is known to be defective in PD [34]. Sentence comprehension taken over all sentence conditions may thus be correlated with the ability to sustain attention.

To summarize, the present study is the first to study sentence comprehension in Dutch in PD. Sentence comprehension was assessed using a sentence-picture matching task with sentence materials which varied in two ways: (1) phrase structure complexity using active versus passive sentences and (2) sentence length. Based on earlier research, a lower overall sentence comprehension score for the PD patients relative to a healthy control group (matched for age, gender, and education) was expected as well as correlations between sentence comprehension and particular measures of executive functions in patients with PD.

## 2. Materials and Methods

### 2.1. Subjects

Twenty-eight idiopathic PD patients participated in this study. Medical and demographic information for this group is given in Table 1. PD patients were diagnosed according to the criteria of the United Kingdom Parkinson's Disease Society Brain Bank. They were assessed with the unified parkinson's disease rating scale, part III (UPDRS) [35]. The UPDRS score was used to estimate the Hoehn and Yahr (H&Y) stage [36] averaged over the best and worst condition per patient. According to the H&Y disability staging criteria, the average motor disability of PD patients included in this study ranged from mild to moderate. A Levodopa equivalent daily dose score (LEDD-score) was calculated for all patients according to the following formula: regular Levodopa dose × 1 + slow release Levodopa ×  .75 + bromocriptine × 10 + apomorphine × 10 + ropinirole × 20 + pergolide × 100 + pramipexole × 100 + (regular Levodopa dose + (slow release Levodopa ×  .75)) ×  .2 if taking entacapone [37]. All patients were assessed while on medication. On the basis of the medication schedule, we chose the optimal moment of the day to conduct the experiment. To prevent wearing-off effects during the assessments, a break was included during which patients could take their anti-Parkinsonian medication and could rest.

PD patients were matched for age, gender, and education with 28 healthy control subjects, who were recruited from the Groningen community (see Table 1). Healthy participants were screened for any history (present as well as past) of neurological or psychiatric conditions by means of a semi-structured interview prior to inclusion. Exclusion criteria were dementia (minimental state examination (MMSE) < 25) [38] and depression (Montgomery-Åsberg depression rating scale (MADRS) [39] ≥ 18) [40]. Candidates using medication that might have affected task performance were also excluded. Patients and healthy control subjects were all native speakers of Dutch, who reported no premorbid language difficulties (such as dyslexia) and had self-reported normal or corrected-to-normal vision and hearing. The Medical Ethical Committee of the University Medical Center Groningen (UMCG) approved this study. Prior to study inclusion, all participants gave their written informed consent according to the Declaration of Helsinki.

### 2.2. Sentence Materials

Ten transitive verbs were selected and used to form 80 sentences, each describing an event. The verbs were controlled for lemma frequency and transitivity according to the Dutch Celex database [41].

Active and passive structures of two lengths were tested: long and short active and long and short passive sentences (each condition consisting of 20 items). The length manipulation involved the inclusion of an adjective before the agent and theme nouns and inclusion of a modifying prepositional phrase (PP) following the agent and theme (see (1) and (2) for an example of the length manipulation). The sentences were semantically reversible (e.g., “*The horse is kicking the cow*”*/*“*The cow is kicking the horse*”) so that participants could not use real-world knowledge to aid in comprehension. The complete list of Dutch sentences per condition and their English translation is provided in Appendix A.

1. Short active sentence
2. De jongen duwt het meisje.
3. (“*The boy pushes the girl*”).

(2) Long active sentence

  De *ouderwetse* jongen met het *gestreepte* hemd duwt het *modieuze* meisje *met de drukke jurk. *

  (“*The old-fashioned boy in the striped shirt pushes the fashionable girl in the printed dress*”).

This resulted in four versions of each sentence which were presented with congruent and incongruent pictures, giving eight combinations altogether.

For 20 sentences incongruence with the picture was based on a role reversal (i.e., agent-patient relationship in the sentence was reversed relative to the picture) and for the other 20 sentences incongruence was lexical (i.e., action was not described by the verb used in the sentence). 

**Figure 1:**
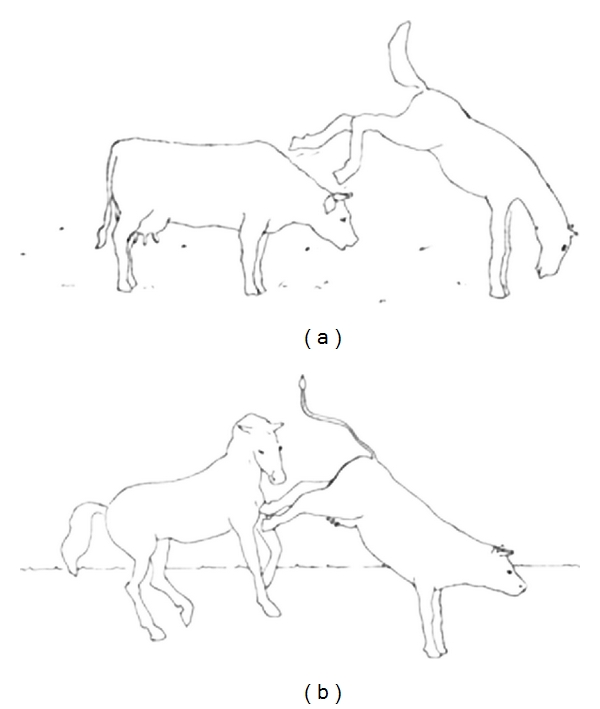
(a) and (b) comprise a picture pair. (a) shows a short active sentence congruent with the picture. (b) is an illustration of a short passive sentence that is incongruent with the picture. A translation in English is given in italics.

A standard “picture-verification task” was used to test comprehension of the sentences. Ten pictures were paired with ten pictures in which the agent and theme were reversed (see Figure 1). Thus, in total, twenty different black-and-white line drawings were shown on a computer screen and each drawing depicted an action involving two characters (the agent and theme of the action). The participant was instructed to determine whether or not the picture was congruent with a spoken sentence presented by the experimenter. Half of the spoken sentences were congruent with the pictures, the other half were not. Each picture was presented four times, resulting in a total of 80 items. The order of sentences was pseudorandom and was the same for each subject.

### 2.3. Neuropsychological Assessment

Two subtests of the testbattery of attentional performances (TAP) [42] were administered to assess sustained visual attention and sustained auditory attention. The sustained visual attention task lasted 10 minutes during which participants had to push a button when they detected irregularities in a regular pattern of movement of an object on a computer screen. The number of times participants did not recognize an irregularity was recorded. The sustained auditory attention task also had a duration of 10 minutes and consisted of a regular sequence of high and low tones. The participants had to detect irregularities in the sequence and the number of undetected irregularities was counted.

The trail making test parts A and B (TMT A & B) [43] and the odd man out test (OMO-Test) [44] were administered to assess set switching. The target measure of the Trailmaking was the performance on part B corrected for psychomotor speed (by dividing it by the performance on part A): the B/A index. The total error score was the target measure of the OMO-Test.

The Stroop task [45] was used to assess inhibition. The target measure was the time score on the Stroop-Color-Word Card divided by the time needed on the Stroop-Color Card, which also corrected for psychomotor speed.

The digit span of the Wechsler adult intelligence scale (WAIS) [46] was used to assess verbal short-term and verbal working memory. A series of digits are read to the participants, who are required to repeat the digits either in the given order (forward condition) or in the reverse order (backward condition). The total number of strings that is repeated correctly is recorded.

Finally, cognitive sequencing was evaluated with a protocol based on Lelekov et al. [47] (see also [48] for a detailed description of the test adapted for Dutch). The aim of the task was to assess the ability to learn letter-sequences with a complex abstract structure (e.g., A-B-C-B-A-C) in order to judge whether a given letter sequence followed the pattern or not. The assessment was terminated if participants showed a persistent inability to perform the task. Uncompleted testing was scored at chance level (i.e., score of 10/20). 

### 2.4. Procedures

The sentence comprehension experiment started with a practice session of four sample sentences. During this practice session, the experimenter corrected errors to ensure the participant understood the task sufficiently. The participants were seated in front of a computer screen and were presented with the black-and-white line drawings, one at a time. The participants were instructed to listen to the sentence read by the experimenter and then to decide whether the sentence was congruent with the picture or not. The sentence was repeated once if the participant asked. Participants' responses were manually recorded during the task. Feedback about the accuracy of responses was not provided.

All participants completed the sentence comprehension test as part of a larger language test battery that was conducted during a single session in a silent room. Participants were given breaks in between tests. The neuropsychological data were collected in a separate session (on the same day). For half of the participants of each group, these preceded the language session, for the other half, they followed the language session. 

### 2.5. Statistical Analyses

One of the sentence items, an incongruent long active sentence, turned out to be ambiguously constructed and was, therefore, scored as correct for all participants (we checked if the interaction effects changed when removing the ambiguous item from the eight sentence conditions. The results of this analysis showed the same pattern as the analysis reported here (see Appendix C)).

The design used in this experiment is a 2 (syntactic complexity) × 2 (length) × 2 (congruence) within-subjects' repeated measures ANOVA design. Not all variables were normally distributed. The assumption of homogeneity of variance was violated as well. In an attempt to avoid these problems, we carried out an arcsine transformation on the results scored as proportion correct, but the assumptions on which the ANOVA depends were still violated. Therefore, nonparametric statistical tests were used. Since Friedman's test for ranks (nonparametric equivalent of a one-way ANOVA) does not allow the examination of interaction effects, a method for calculating the main and interaction effects which can then be tested nonparametrically is employed in this study (for a detailed description see Appendix B). This approach maintains the orthogonality of the design as in an ANOVA, which means that comparisons are independent of each other. Consequently, it avoids the problems of multiple comparisons without making unwarranted assumptions [49].

The total sentence comprehension score of PD patients and healthy control subjects was compared using the Mann-Whitney *U*-test. Main effects and interaction effects between the different manipulations within the sentence comprehension task (phrase structure complexity, sentence-length and picture-sentence congruence, and the interactions of these factors) were tested using the Wilcoxon signed-rank test (for the calculations see Appendix B). 

Turning to the cognitive tests, the performance of the PD patients and healthy control subjects was compared on all cognitive tests using a Mann-Whitney *U*-test. Since the TMT and the OMO test were both used to assess set switching, the scores on these tests were combined using z-scores. Finally, hypothesis-driven (one-tailed; see introduction) Spearman correlations between the total scores and subscores on the sentence comprehension task and the scores on the cognitive tests of the PD patients were computed. 

## 3. Results

### 3.1. Comparison between Groups on Sentence Comprehension Task

Table 2 summarizes the performance of the healthy control subjects and PD patients on the eight conditions of the sentence comprehension task. Overall, PD patients scored significantly lower (median = 77, interquartile range (IR) = 4.5) than healthy control subjects (median = 78, IR = 4) on the total sentence comprehension task (*Z* = −2.05, *P* = .04). 

### 3.2. Main and Interaction Effects of Linguistic Variables and Group

Comparison of the scores for congruent and incongruent items revealed that for both groups combined, the incongruent items were significantly more difficult than the congruent items (*Z* = −3.045, *P* = .002). There was no interaction between group and congruence (*Z* = −.025, *P* = .980). Neither PD participant's accuracy score (*Z* = −.179, *P* = .858) nor healthy control subjects' accuracy score (*Z* = −.194, *P* = .846) showed any effect of the type of violation (lexical violation or role reversal) when incongruent items were tested separately. Therefore, the lexically violated and role reversal items were combined per condition for further analyses.

In addition to the main effect for congruence, a main effect of syntactic complexity was also found for both groups combined; that is, comprehension of passive sentences was more difficult than that for the active sentences (*Z* = −4.875, *P* < .001). There was no difference for the effect of syntactic complexity between the two groups (*Z* = −.835, *P* = .404). Comparison of the scores for the two groups combined for sentence length showed no effect of length (*Z* = −1.394, *P* = .163), which again did not differ between groups (*Z* = −.51, *P* = .610). 

To evaluate the interactions between congruence, syntactic complexity, and length, new variables were computed for both groups combined (see Appendix B).  Table 3 illustrates the interaction effects (for both groups combined and for difference between groups) and shows the scores of both PD patients and healthy control subjects.

A significant interaction effect of congruence by length was found for the two groups combined which also did not differ between the groups (see Table 3). Within the incongruent items, the long items were more difficult than the short items (*Z* = −3.632, *P* < .001). However, within the congruent items no difference was found between processing of the short and long items (*Z* = −1.578, *P* = .155). 

No interaction effect of syntactic complexity and length was found for the two groups combined, which also did not differ between the groups. In addition, no interaction effect of syntactic complexity and congruence was observed in the two groups combined, and no difference was found between the groups (see Table 3).

For both groups combined, the interaction between length and syntactic complexity differed significantly for the congruent as opposed to the incongruent items. Again, no difference was found between the groups (see Table 3). Within the incongruent item group the long passive sentences were more difficult to comprehend than the short passive sentences (*Z* = 4.309, *P* < .001). 

To summarize, there were a number of significant effects of the manipulations of complexity, congruence, and length, but none of these showed any interaction with group, suggesting the groups had the same pattern of results. To confirm that both groups independently showed each of the significant patterns implied by the main effect, we did the comparisons for each group alone as well and found that indeed both groups showed each of these effects either significantly or with a strong tendency to significance. This confirms that both groups show the same pattern of errors.

### 3.3. Comparisons between Groups on Cognitive Measures

Compared to healthy control subjects, PD patients scored significantly lower on set switching and on sustained visual attention (see Table 4). For all other cognitive functions no significant differences were found between groups. 

It is important to mention is that four PD patients were unable to perform the complex sequencing task because of difficulties with task comprehension. Their testing was scored at chance level (i.e., score of 10/20). 

### 3.4. Correlation Analyses

A Bonferroni correction was applied to correct for multiple correlations using an adjusted significant level of  .008 (i.e.,   .05 divided by 6 (number of correlations). The expectation that the total sentence comprehension score would correlate with sustained attention in PD patients was confirmed with a negative correlation between the total sentence comprehension score and sustained visual attention score (*r*_*s*_ = −.474, *P* = .006). A correlation between set switching and the comprehension of passive sentences (*r*_*s*_ = −.53, *P* = .002) was found in PD patients. The passive sentences did not correlate significantly with the digit span backward score (*r*_*s*_ = .335, *P* = .041) or the measure of inhibition in PD (*r*_*s*_ = −.282, *P* = .077). No correlation was found (*r*_*s*_ = .234, *P* > .05) between the performance on the complex abstract structure sequencing task and the comprehension of passive sentences in PD patients. Finally, working memory and length were also predicted to correlate. However, since no effect of length was found, correlations with the short-term memory and the long sentences were not conducted. 

The theories discussed in the introduction suggest these correlations should be specific to performance on passive sentences. To confirm this, we checked to make sure that none of these neuropsychological scores correlated with performance on active sentences and failed to find any trend toward significance in these correlations.

According to Salthouse [50] several forms of effortful cognitive processing continue to decline throughout adulthood. It is, therefore, possible that sentence comprehension was influenced by declining executive functions in healthy older subjects too. On the basis of the similar patterns found in the two groups for the sentence comprehension and the executive function tasks, we further examined the relations between the executive functions scores and sentence comprehension. First, we conducted the same correlation analyses in the healthy adults as a group. Second, both groups were combined to conduct the same analyses (also in this case the Bonferroni corrected significance level of  .008 was used). 

No significant correlation between sentence comprehension and cognitive functions was found for the analysis in the healthy control group. The correlations for both groups combined were as follows: sustained visual attention (*r*_*s*_ = −.490, *P* < .001), sustained auditory attention (*r*_*s*_ = −.205, *P* = .153), digit span backward (*r*_*s*_ = .309, *P* = .010), Set switching (*r*_*s*_ = −.421, *P* = .001), interference index Stroop (*r*_*s*_ = −.024, *P* = .432), and complex sequencing complex (*r*_*s*_ = .286, *P* = .017).

## 4. Discussion

This study investigated the functional basis of sentence comprehension deficits in PD patients. We assessed the comprehension of active and passive sentences that varied in length in 28 Dutch-speaking PD patients and age-, gender-, and education-matched healthy control subjects. Associations were determined between sentence comprehension and relevant executive functions.

PD patients showed significantly decreased overall sentence comprehension compared to healthy control subjects. However, this impairment was fairly mild, consistent with earlier reports [4, 5]. Additionally, cognitive functions other than sentence comprehension were also relatively intact. While PD patients showed decreased performance on the visual attention and set-switching tests, other cognitive functions did not differ significantly from those of healthy controls. It should be noted that the PD group examined in this study had mild-to-moderate motor disability and were not depressed or demented. Cognitive function declines with the progression of the disease [51]. PD patients' working memory, inhibition and cognitive sequencing abilities, as well as comprehension performance would probably be poorer in a more severely affected group of patients. Our results support the claim that even in relatively mild PD, some sentence comprehension deficits are present. Overall, our results emphasize that the simple structures contribute to the effect as well as the more complex ones.

The language factors produced virtually identical patterns in both groups as well. As in previous studies, both PD patients and healthy control subjects showed more difficulties with noncanonical syntactic structure (i.e., the passive). However, the length manipulation used here did not significantly affect sentence comprehension in either group, as no difference in comprehension was found between the long and short sentences. Apparently, the memory demands associated purely with length were not particularly burdensome. This finding is consistent with the results of Grossman et al. [21] who found that mild PD patients showed comparable performance patterns on the behavioural sentence comprehension task relative to a control group. Skeel et al. [23] contrasted semantically and syntactically loaded sentences of several levels of difficulty. Comparable to the PD patients included in the present study, these patients did not show deficits in working memory. However, in contradiction to our findings, they demonstrated deficits in processing both syntactically and semantically loaded sentences. 

A main effect of picture congruence was found, and an interaction effect between congruence, complexity, and length was also evident in both groups. This suggests that the processing of incongruent items is more difficult than the processing of congruent items, particularly for long, complex (i.e., passive voice) sentences. Thus, both PD patients and healthy participants showed more difficulties with long passive sentences when they were incongruent with visually presented nonlinguistic information against which they had to be verified. Long passives included two prepositional phrases (following and modifying each of the nouns in the sentence). This may have relevance to why long passive sentences are particularly more difficult than long active sentences in the more cognitive demanding incongruent trials, as the information contained in these phrases was not very useful in judging congruence.

Some researchers suggest that PD leads to specific deficits in language processing, like in aphasia [6–9]. This is not consistent with our findings. Although we found several main effects (syntactic complexity and congruence) and interactions (congruence with length and congruence with length and complexity) in the analyses including both groups, none of the effects differed significantly between the two groups. Separately, these effects were significant or neared significance for both groups, demonstrating that both groups showed the same pattern of results. Some previous studies found a clear effect of complexity (e.g., [11, 12, 52]). However, these studies mostly investigated center and final embedded sentences instead of simple passives like in the present study. PD patients seem to have problems with switching sets at boundaries between clauses [11, 12]. These impairments for passive sentences were mostly found in PD when combined with another difficulty-inducing factor [10–12]. It might thus be concluded that PD does not have a specific effect on the processing of passive sentences and that PD influences more the processing of grammatical complexity of embedded sentences. Deficits in the processing of the passives may instead be associated with a more nondomain-specific executive deficit. 

This point is related to the issue of the specificity within the executive function effects. Those factors which were significantly impaired, even in this mild group, correlated with deficits, suggesting that with the progression of the disease more executive deficits and related sentence comprehension deficits will likely occur. For healthy controls, no significant associations were found between sentence comprehension and executive functions. Wilson et al. [53] found that as a group, older adults declined in their cognitive performance over time though wide individual differences were evident at all ages. This great variability among healthy individuals might explain why in the present study we did not find any association between the scores of the healthy controls on sentence comprehension measures and executive functions tasks. 

Furthermore, although the decline in executive functions in healthy individuals resembles the impairments in executive functions found in PD, the decline is not as pronounced as in PD patients. This might explain why we found associations between visual attention, set switching, and sentence comprehension in both groups combined that were comparable to the associations we found in PD patients. The PD patients in our study showed deficits in sustained visual attention relative to the healthy control subjects, which was associated with a decreased total sentence comprehension score. The picture-verification task used in the present study is obviously an attention-demanding task and includes the simultaneous use of two sensory stimulus modalities. Impaired visual sustained attention might have interfered with the comparison of the auditory sentence representation and the visual input and thus explain the relation found between PD patients' total sentence comprehension score and the sustained visual attention score. This correlation is not relevant for comprehension per se but points to the effects of task demands on PD patients' performance during a picture-verification task. The comprehension of passive sentences of PD patients in our study was associated with impaired set switching, similar to the data of Hochstadt [11, 24] and Hochstadt et al. [12]. Since comprehending a sentence demands that a listener flexibly guides his/her attention to relevant linguistic information, it can be suggested that impaired set switching contributes to the difficulties some PD patients have comprehending passive sentences.

Working memory has also been claimed to be associated with comprehension of syntactically complex sentences, such as passive sentences in healthy individuals. Consistent with this hypothesis, the PD patients showed an association with the manipulation of information in working memory, as assessed by backward digit span, and comprehension of passive sentences. Although this result became nonsignificant after Bonferroni correction, we suggest conducting future research on the relation between the manipulative aspects of working memory and the comprehension of passive sentences in PD patients.

Language places high demands on cognitive sequence processing, as it requires processing according to rules [29, 30]. However, in PD patients, no association between the complex sequencing task and the comprehension of passive sentences was found. A possible explanation for the lack of this association in PD lies in the fact that some PD patients were unable to perform the complex sequencing task, leading to too low a variance to determine the effects of sequencing ability. The associations found between PD patients' sentence comprehension of passive sentences and specific executive functions thus offer additional evidence to the assumption that executive functions interact with the language faculty while comprehending syntactically complex sentences [7, 10–16, 24, 25] and that impairments of these functions lead to comprehension deficits rather than a language-specific impairment. The evidence suggests that several executive functions are involved during sentence comprehension. 

The maintenance of information in short-term memory was expected to be related to the length manipulation. However, neither group demonstrated a difference between the processing of long and short sentences. These results suggest that the extra memory load due to length did not lead to impaired comprehension.

Even though the current study adds important insights to the underlying nature of the sentence comprehension deficits in Dutch-speaking PD patients, two issues need to be addressed in future research. First, the PD patients in this study were assessed while on their regular medications. In order to maintain patients' quality of life and to avoid frustration and confounds due to increased motor symptoms. However, according to the overdose hypothesis [54, 55], dopaminergic medication can selectively impair cognitive functions that are served by a basal ganglia region that is relatively spared, as well as enhancing the cognitive functions of a dysfunctional basal ganglia region. Grossman et al. [56] found that PD patients' sentence comprehension worsened while they were off their dopaminergic medication. Therefore, future research will need to explore the relationships between dopaminergic medications, sentence comprehension, and executive functions. Secondly, the off-line measure used in this study is somewhat limited. The present findings should be replicated using on-line measures of sentence comprehension in order to disentangle the impact of executive functions necessary for syntactic processes per se and the executive functions necessary for the sentence comprehension task.

## 5. Conclusions

The present findings confirmed previous results from other labs demonstrating a sentence comprehension deficit even in the mild to moderate PD patients. However, the results obtained do not support the claim that the difficulties encountered by patients with PD are limited to one aspect of sentence processing (i.e., noncanonical or long structures). Instead, the patients showed a more nonspecific comprehension impairment, and the pattern of errors was identical with that found in the healthy control subjects. The sentence comprehension deficits appear to be dependent on executive function. Decreased set-switching abilities were associated with comprehension of noncanonical passive sentences. Deficits in sustained visual attention appear to underlie the overall decrement in performance, possibly due to the demands of the sentence-picture matching task. Generally, our study confirms that there does not appear to be an encapsulated language faculty which functions independent from executive functioning.

## Figures and Tables

**Table 1:** Demographic and clinical features (mean (±SD)) of the PD patients and healthy control subjects.

	PD patients	HC subjects
	(*n* = 28)	(*n* = 28)
Gender (male : female)	16 : 12	16 : 12
Handedness (right : left)	27 : 1	24 : 4
Age in years	61.39 (8.80)	62.93 (9.04)
Education in years	13.21 (3.90)	13.57 (3.25)
MMSE score	28.11 (1.13)	27.64 (1.22)
MADRS score	6 (4.00)	3.32 (2.83)
Duration of disease in years	6.04 (4.55)	n/a
UPDRS part III	15.68 (5.35)	n/a
(i) Right-side hypokinesia	3.84 (2.43)	n/a
(ii) Left-side hypokinesia	3.12 (2.28)	n/a
H&Y staging	1.79 (.52)	n/a
LEDD-score	786.94 (472.45)	n/a

HC: healthy control, n/a: not applicable.

**Table 2:** Scores correct (median and interquartile range) for the eight sentence comprehension conditions, obtained for the PD patients and healthy control subjects.

Sentence variables			PD patients		HC subjects	
Congruence	Synt. Compl.	Length	Median	IR	Median	IR
Congruent	Actives	Short	10	0	10	0
		Long	10	1	10	0
	Passives	Short	10	1	10	1
		Long	10	1	10	0
Incongruent	Actives	Short	10	1	10	1
		Long	10	0	10	0
	Passives	Short	10	1	10	0
		Long	9	2	9	1

The maximum possible per condition is 10. HC: healthy control, Synt. Compl.: syntactic complexity, IR: interquartile range.

**Table 3:** The interaction effects for the two groups combined and the differences for the interaction variables between the groups. Scores correct (median and interquartile range) for sentence comprehension of specific sentence types, obtained for the PD and healthy control subjects.

Interaction variables	PD patients	HC subjects	Both groups combined		Differences between groups	
Sentence types	Median (IR)	Median (IR)	*Z*	*P*	*Z*	*P*
Congruence × length			−3.233	.001*	−.547	.584

Congruent short	19 (2)	20 (1)				
Congruent long	20 (1)	20 (0)				
Incongruent short	19 (1)	20 (1)				
Incongruent long	19 (2)	19 (1)				

Syntactic complexity × length			−1.341	.180	−.1	.920

Active short	20 (1)	20 (1)				
Active long	20 (1)	20 (0)				
Passive short	20 (2)	20 (1)				
Passive long	19 (1)	19 (2)				

Congruence × syntactic complexity			−.698	.485	−.506	.613

Congruent active	20 (1)	20 (0)				
Congruent passive	19 (2)	20 (1)				
Incongruent active	19.5 (1)	20 (1)				
Incongruent passive	18.5 (2)	19 (2)				

Congruence × length × syntactic complexity			−4.692	.001*	−.55	.583

The maximum possible per sentence type is 20. Statistical significance is indicated as follows: *P ≤ .001. See Appendix B for a detailed description of the procedures to calculate the scores for the different sentence types.

HC: healthy control, IR: Interquartile Range.

**Table 4:** Neuropsychological performance measures (median and interquartile range) obtained for the healthy control subjects compared to the PD patients.

Neuropsychological tests	PD patients		HC subjects		PD ≠ HC	
	Median	IR	Median	IR	*Z*	*P*
Sustained visual attention	0	2	0	0	−3.055	.002**
Sustained auditory attention	0	1	0	1	−.747	.455
Digit span backward	7	4	7	3	−.803	.422
Set switching (TMT A & B; OMO test)	−.01	1.19	−.35	.64	−2.518	.012*
Interference index Stroop	1.61	.43	1.54	.45	−.977	.329
Sequencing Complex (max = 20)	19	2	19	2	−.782	.434

Statistical significance is indicated as follows: *P ≤ .05, **P ≤ .01.

HC: healthy control.

**Table 5:** The interaction effects for the two groups combined and the differences for the interaction variables between the groups, when the ambiguous item “redden” (to save) was removed from the dataset. Scores correct (median and interquartile range) for sentence comprehension of specific sentence types, obtained for the PD and healthy control subjects.

Interaction variables	PD patients	HC subjects	Both groups combined		Differences between groups	
Sentence types	Median (IR)	Median (IR)	*Z*	*P*	*Z*	*P*
Matching × length			−3.182	.001*	−.440	.660

Congruent short	17.5 (1)	18 (1)				
Congruent long	18 (1)	18 (0)				
Incongruent short	18 (1)	18 (0)				
Incongruent long	17 (1.75)	18 (1)				

Syntactic complexity × length			−.390	.697	−.405	.686

Active short	18 (.75)	18 (0)				
Active long	18 (1)	18 (0)				
Passive short	18 (2)	18 (1)				
Passive long	17 (1.75)	18 (1)				

Matching × syntactic complexity			−.939	.348	−1.113	.266

Congruent active	18 (1)	18 (0)				
Congruent passive	17 (1.75)	18 (1)				
Incongruent active	18 (.75)	18 (0)				
Incongruent passive	17 (2)	18 (1)				

Matching × length × syntactic complexity			−3.322	.001*	−.291	.771

The maximum possible per sentence type is 18. Statistical significance is indicated as follows: *P ≤ .001. See Appendix B for a detailed description of the procedures to calculate the scores for the different sentence types.

HC: healthy control, IR: interquartile range.

## Appendices

### A. Sentence Materials

#### A.1. Congruent Sentences

Active Short (AS)

1. De charmante man met het nieuwe kostuum filmt de goedgeklede vrouw met de rieten mand.
2. The charming man in the new suit videotapes the well-dressed woman with the woven basket.
3. De goed getrainde vrouw met het korte haar draagt de sterk vermagerde man met de scheiding in het haar.
4. The well-trained woman with the short hair carries the extremely emaciated man with the separation in the hair.
5. De verwaarloosde kat met de hongerige maag achtervolgt de nietsvermoedende hond met de korte staart.
6. The neglected cat with the hungry stomach chases the unsuspecting dog with the short tail.
7. Het verlegen meisje met de staartjes kust de geschrokken jongen met het witte hemd.
8. The timid girl with the pigtails kisses the anxious boy in the white shirt.
9. De chic geklede vrouw met de gouden juwelen groet de plechtig uitgedoste man met de hoge hoed.
10. The stylishly dressed woman with the golden jewelry greets the formally dressed man in the top hat.
11. Het boosaardige meisje met de gekruiste armen bewaakt de machteloze jongen met de korte broek.
12. The malicious girl with the crossed arms guards the defenseless boy in the shorts.
13. De ouderwetse jongen met het gestreepte hemd duwt het modieuze meisje met de drukke jurk.
14. The old-fashioned boy in the striped shirt pushes the fashionable girl in the printed dress.
15. De moedige man met de opgerolde mouwen redt de verdrinkende vrouw met de doorweekte kleren.
16. The brave man with the rolled-up sleeves saves the drowning woman in the drenched clothes.
17. Het zelfstandige kind met de kleine staartjes wast de hulpeloze vrouw met het verdrietige gezicht.
18. The independent child with the small pigtails washes the helpless woman with the sad face.
19. De hevig tekeergaande koe met de hoorns schopt het erg angstige paard met de trillende benen.
20. The violently charging cow with the horns kicks the very anxious horse with the trembling legs.

Passive Short (PS)

1. De vrouw wordt door de man gefilmd.
2. The woman is videotaped by the man.
3. De man wordt door de vrouw gedragen.
4. The man is carried by the woman.
5. De hond wordt door de kat achtervolgd.
6. The dog is chased by the cat.
7. De jongen wordt door het meisje gekust.
8. The boy is kissed by the girl.
9. De man wordt door de vrouw gegroet.
10. The man is greeted by the woman.
11. De jongen wordt door het meisje bewaakt.
12. The boy is guarded by the girl.
13. Het meisje wordt door de jongen geduwd.
14. The girl is pushed by the boy.
15. De vrouw wordt door de man gered.
16. The woman is saved by the man.
17. De vrouw wordt door het kind gewassen.
18. The woman is washed by the child.
19. Het paard wordt door de koe geschopt.
20. The horse is kicked by the cow.

Passive Long (PL)

1. De goedgeklede man met het nieuwe kostuum wordt door de charmante vrouw met de lichte zomerjurk gefilmd.
2. The well-dressed man in the new suit is videotaped by the charming woman in the light summer dress.
3. De licht gekwetste vrouw met de verzwikte enkel wordt door de goed getrainde man met de nieuwe schoenen gedragen.
4. The slightly hurt woman with the twisted ankle is carried by the well-trained man with the new shoes.
5. De nietsvermoedende kat met de lange staart wordt door de achtergelaten hond met de hongerige maag achtervolgd.
6. The unsuspecting cat with the long tail is chased by the abandoned dog with the hungry stomach.
7. Het zeer opgewekte meisje met de bolletjestrui wordt door de erg verlegen jongen met de blozende kaken gekust.
8. The very bright girl in the dotted pullover is kissed by the very timid boy with the blushing cheeks.
9. De onzekere vrouw met het opgestoken haar wordt door de welbespraakte man met de opvallende das gegroet.
10. The insecure woman with the updo is greeted by the eloquent man with the striking tie.
11. Het machteloze meisje met de vastgebonden handen en voeten wordt door de boosaardige jongen met het speelgoedgeweer bewaakt.
12. The defenseless girl with the tied hands and feet is guarded by the malicious boy with the toy rifle.
13. De ouderwetse jongen met de korte broek wordt door het modieuze meisje met de drukke jurk geduwd.
14. The old-fashioned boy in the shorts is pushed by the fashionable girl with the printed dress.
15. De verdrinkende man met de doorweekte kleren wordt door de moedige vrouw zonder schoenen gered.
16. The drowning man in the drenched clothes is saved by the brave woman without shoes.
17. Het ongehoorzame kind met de smerige handen wordt door de vermoeide vrouw met het korte haar gewassen.
18. The disobedient child with the dirty hands is washed by the tired woman with the short hair.
19. De angstige koe met de hoorns wordt door het ongetemde paard met de krachtige benen geschopt.
20. The anxious cow with the horns is kicked by the untamed horse with the powerful legs.

#### A.2. Incongruent Sentences

Active Short (AS): Lexical

1. De vrouw groet de man.
2. The woman greets the man.
3. De hond bijt de kat.
4. The dog bites the cat.
5. De jongen volgt het meisje.
6. The boy follows the girl.
7. De vrouw bewaakt de man.
8. The woman guards the man.
9. De vrouw slaat het kind.
10. The woman hits the child.

Active Short (AS): Role Reversal

1. De vrouw draagt de man.
2. The woman carries the man.
3. Het meisje kust de jongen.
4. The girl kisses the boy.
5. De vrouw groet de man.
6. The woman greets the man.
7. De jongen duwt het meisje.
8. The boy pushes the girl.
9. De koe schopt het paard.
10. The cow kicks the horse.

Active Long (AL): Lexical

1. De charmante man met het nieuwe kostuum groet de goedgeklede vrouw met de rieten mand.
2. The charming man in the new suit greets the well-dressed woman with the woven basket.
3. De goed getrainde vrouw met het korte haar slaat de sterk vermagerde man met de scheiding in het haar.
4. The well-trained woman with the short hair beats the extremely emaciated man with the separation in the hair.
5. Het verlegen meisje met de staartjes draagt de geschrokken jongen met het witte hemd.
6. The timid girl with the pigtails carries the anxious boy in the white shirt.
7. De chic geklede vrouw met de gouden juwelen bedreigt de plechtig uitgedoste man met de hoge hoed.
8. The stylishly dressed woman with the golden jewelry threatens the formally dressed man with the top hat.
9. De ouderwetse jongen met het gestreepte hemd kust het modieuze meisje met de drukke jurk.
10. The old-fashioned boy in the striped shirt kisses the fashionable girl in the printed dress.

Active Long (AL): Role Reversal

1. De nietsvermoedende hond met de korte staart achtervolgt de verwaarloosde kat met de hongerige maag.
2. The unsuspecting dog with the short tail chases the neglected cat with the hungry stomach.
3. De machteloze jongen met de korte broek bewaakt het boosaardige meisje met de gekruiste armen.
4. The defenseless boy with the shorts guards the malicious girl with the crossed arms.
5. De verdrinkende vrouw met de doorweekte kleren redt de moedige man met de opgerolde mouwen.
6. The drowning woman in the drenched clothes saves the brave man with the rolled-up sleeves.
7. De hulpeloze vrouw met het verdrietige gezicht wast het zelfstandige kind met de kleine staartjes.
8. The helpless woman with the sad face washes the independent child with the small pigtails.
9. Het erg angstige paard met de trillende benen schopt de hevig tekeergaande koe met de hoorns.
10. The very anxious horse with the trembling legs kicks the violently charging cow with the horns.

Passive Short (PS): Lexical

1. De vrouw wordt door de man gegroet.
2. The woman is greeted by the man.
3. De man wordt door de vrouw geslagen.
4. The man is beaten by the woman.
5. De jongen wordt door het meisje gedragen.
6. The boy is carried by the girl.
7. De man wordt door de vrouw bedreigd.
8. The man is threatened by the woman.
9. Het meisje wordt door de jongen gekust.
10. The girl is kissed by the boy.

Passive Short (PS): Role Reversal

1. De kat wordt door de hond achtervolgd.
2. The cat is chased by the dog.
3. Het meisje wordt door de jongen bewaakt.
4. The girl is guarded by the boy.
5. De man wordt door de vrouw gered.
6. The man is saved by the woman.
7. Het kind wordt door de vrouw gewassen.
8. The child is washed by the woman.
9. De koe wordt door het paard geschopt.
10. The cow is kicked by the horse.

Passive Long (PL): Lexical

1. De goedgeklede man met het nieuwe kostuum wordt door de charmante vrouw met de lichte zomerjurk gegroet.
2. The well-dressed man in the new suit is greeted by the charming woman in the light summer dress.
3. De nietsvermoedende kat met de lange staart wordt door de achtergelaten hond met de hongerige maag gebeten.
4. The unsuspecting cat with the long tail is bitten by the abandoned dog with the hungry stomach.
5. Het machteloze meisje met de vastgebonden handen en voeten wordt door de boosaardige jongen met het speelgoedgeweer gevolgd.
6. The defenseless girl with the tied hands and feet is chased by the malicious boy with the toy rifle.
7. De verdrinkende man met de doorweekte kleren wordt door de moedige vrouw zonder schoenen bewaakt.
8. The drowning man in the drenched clothes is guarded by the brave woman without shoes.
9. Het ongehoorzame kind met de smerige handen wordt door de vermoeide vrouw met het korte haar geslagen.
10. The disobedient child with the dirty hands is beaten by the tired woman with the short hair.

Passive Long (PL): Role Reversal

1. De goed getrainde man met de nieuwe schoenen wordt door de licht gekwetste vrouw met de verzwikte enkel gedragen.
2. The well-trained man with the new shoes is carried by the slightly hurt woman with the twisted ankle.
3. De erg verlegen jongen met de blozende kaken wordt door het zeer opgewekte meisje met de bolletjestrui gekust.
4. The very timid boy with the blushing cheeks is kissed by the very bright girl with the dotted pullover.
5. De welbespraakte man met de opvallende das wordt door de onzekere vrouw met het opgestoken haar gegroet.
6. The eloquent man with the striking tie is greeted by the insecure woman with the updo.
7. Het modieuze meisje met de drukke jurk wordt door de ouderwetse jongen met de korte broek geduwd.
8. The fashionable girl in the printed dress is pushed by the old-fashioned boy in the striped shirt.
9. Het ongetemde paard met de krachtige benen wordt door de angstige koe met de hoorns geschopt.
10. The untamed horse with the powerful legs is kicked by the anxious cow with the horns.

### B. Statistical Analyses

Since Friedman's test for ranks (nonparametric equivalent of ANOVA) does not allow the examination of interaction effects, a method for calculating the main and interaction effects which can then be tested nonparametrically is proposed in this study. The approach described below maintains the orthogonality (i.e., independence of comparisons) of the design as in an ANOVA [49].

#### B.1. Main Effects of the Sentence Variables

First, a total score for correctly congruent and incongruent items was calculated per participant. Then the Wilcoxon Signed-Rank test was used to compare the congruent and incongruent scores of the two groups combined. Thirdly, the incongruent items were subtracted from the congruent items. A Mann-Whitney *U*-test was used to determine if there was a difference between the groups on this variable.

The same procedure was followed to determine the main effects of syntactic complexity and length. In order to do so, a total score for actives and passives and short and long sentences was calculated, respectively.

#### B.2. Interaction Effects between Sentence Variables

To evaluate the interactions between matching, syntactic complexity, and length, new interaction variables were computed for both groups combined.

##### B.2.1. Analysis of the Interaction Effect between Matching and Length

To evaluate whether the effect of length was different for the congruent items, compared to the incongruent items, two new interaction variables were composed: one for congruent and one for incongruent items.

Within the congruent items the following steps were taken to calculate the new interaction variable.

1. All passive long (PL) and Active long (AL) congruent items were combined as in the formula: PL + AL.
2. All passive short (PS) and active short (AS) congruent items were combined as in the formula: PS + AS.
3. The long and short items were subtracted to compose the new “length by matching” variable: (PL + AL) − (PS + AS).
4. The same calculation steps were followed for the incongruent items resulting in the formula: (InconPL + InconAL) − (InconPS + InconAS).

In further analyses, these two new “length by matching” variables were compared using the Wilcoxon signed-rank test to determine whether within subjects the effect of length differed between the congruent and incongruent items. Finally, it was investigated whether these two new length-interaction variables differed between groups.

To do this, the two “length by matching” variables were subtracted from each other and the Mann-Whitney *U*-test was used to determine whether the interaction between length and matching differed between groups.

##### B.2.2. Analysis of the Interaction Effect between Syntactic Complexity and Length

To evaluate whether the effect of length was different for the active and passive items another two interaction variables were composed by calculating the effect of length separately for actives and passives.

Within the active items the following steps were taken to calculate the new interaction variable.

1. The active long items of the congruent (AL) and incongruent items (InconAL) were combined as in the formula: AL + InconAL.
2. All the active short items of the congruent (AS) and incongruent items (InconAS) were combined as in the formula: AS + InconAS.
3. The long and short items were subtracted to compose the new “length by active voice” variable: (AL + InconAL) − (AS + InconAS).
4. The same calculation steps were followed for the passive items, leading to: (PL + InconPL) − (PS + InconPS).

In further analyses, these length variables were compared using the Wilcoxon signed-rank test to see whether the effect of length differed between the active and passive items for the two groups combined. In addition, it was determined whether the interaction between syntactic complexity and length differed between groups. Therefore, the variables were subtracted and a Mann-Whitney *U*-test was used to calculate the difference between groups.

##### B.2.3. Analysis of the Interaction Effect between Matching and Syntactic Complexity

To evaluate whether the effect of syntactic complexity was different for the congruent and incongruent items, two final interaction variables were composed by subtracting the active and passive items within the congruent and incongruent items separately.

Within the congruent items the following steps were taken to calculate the new interaction variable.

1. All congruent short passives (PS) and long passives (PL) were combined as in the formula: PS + PL.
2. All congruent short actives (AS) and long actives (AL) were combined as in the formula: AS + AL.
3. The passive and active items were subtracted to compose the new “syntactic complexity by congruent” variable: (PS + PL) − (AS + AL).
4. The same calculation steps were followed within the incongruent items resulting in the formula: (InconPS + InconPL) − (InconAS + InconAL).

In further analyses, these two new syntactic complexity variables were compared using the Wilcoxon signed-rank test to see whether all subjects combined showed a difference in the effect of syntactic complexity depending on whether the items congruent the picture or not. To determine whether the interaction between matching and syntactic complexity differed between groups, the congruent variable was subtracted from the incongruent variable. The Mann-Whitney *U*-test was used to investigate the differences between the groups.

##### B.2.4. Analysis of the Three-Way Interaction between Length, Syntactic Complexity, and Matching

Following the same logic described above, the three-way interaction will reveal whether the length versus syntactic complexity interaction differed for the congruent and incongruent items. Using the following formulas, the three-way interaction variable was calculated for the congruent and incongruent items, respectively:

1. (PL − PS) − (*AL*⁡−AS),
2. (InconPL − InconPS) − (InconAL − InconAS).

Subsequently, the interaction variables for the congruent and incongruent items were compared using Wilcoxon's signed-rank test for both groups together. In order to check for differences between groups, the congruent and incongruent items were subtracted resulting in the following formula:

(B.1) $$\begin{array}{cc} & \left[\left(\text{PL}-\text{PS}\right)-\left(\mathrm{AL}-\text{AS}\right)\right]\\ & \;\;-\left[\left(\text{InconPL}-\text{InconPS}\right)-\left(\text{InconAL}-\text{InconAS}\right)\right]. \end{array}$$

Using the Mann-Whitney test, both groups were compared on the three-way interaction variable.

### C. Interaction Effects and Participants' Scores without the Item “Redden” (to Save)

See Table 5.
